# Elevated levels of salivary interleukin-34 in patients suffering from Alzheimer’s disease

**DOI:** 10.1007/s00784-025-06376-4

**Published:** 2025-05-20

**Authors:** Reuben Clark, Ronaldo Lira-Junior, Leif Jansson, Maria Eriksdotter, Marianne Schultzberg, Pirkko Pussinen, Kåre Buhlin, Elisabeth A. Boström

**Affiliations:** 1 Division of Oral diagnostics and surgery, Department of Dental Medicine, Karolinska Institutet, Huddinge, Sweden; 2 Division of Periodontology, Department of Dental Medicine, Karolinska Institutet, Huddinge, Sweden; 3 Center for Alzheimer Research, Division of Clinical Geriatrics, Department of Neurobiology, Care Sciences and Society, Karolinska Institutet, Stockholm, Sweden; 4 Theme Inflammation and Aging, Karolinska University Hospital, Stockholm, Sweden; 5 Center for Alzheimer Research, Division of Neurogeriatrics, Department of Neurobiology, Care Sciences and Society, Karolinska Institutet, Stockholm, Sweden; 6 Department of Oral and Maxillofacial Diseases, University of Helsinki, Helsinki, Finland; 7 Institute of Dentistry, University of Eastern Finland, Kuopio, Finland; 8 Department of Orofacial Medicine, Folktandvården Stockholms Län AB, Stockholm, Sweden

**Keywords:** Saliva, Cerebrospinal fluid, Cognition, Cytokine, Interleukin-34, Periodontal disease

## Abstract

**Objectives:**

To investigate interleukin (IL)-34 and colony-stimulating factor (CSF)-1 levels in saliva, cerebrospinal fluid, and plasma in different stages of cognitive impairment. The study also examines the relationship between these biomarkers and periodontal status across different stages of cognitive impairment.

**Material and Methods:**

A total of 230 individuals diagnosed with Alzheimer’s disease (AD, *n = *52), mild cognitive impairment (MCI, *n = *51), subjective cognitive impairment (SCI, *n = *51), and controls (*n = *76) were enrolled. Participants underwent clinical and radiological oral examinations. Cerebrospinal fluid samples were collected from all groups except controls. Stimulated saliva and blood were collected during oral examination. IL-34 and CSF-1 levels were assessed using enzyme-linked immunosorbent assays.

**Results:**

Salivary IL-34 levels were increased in AD compared to SCI (*p =* 0.010) and controls (*p <* 0.001), and in MCI compared to controls (*p <* 0.001). Elevated salivary CSF-1 levels were observed in AD compared to SCI (*p =* 0.003). Salivary IL-34 was inversely associated with Mini-Mental State Examination (MMSE) scores (*p <* 0.010) and body mass index (*p =* 0.040), while CSF-1 was associated with age (*p =* 0.015). IL-34 and CSF-1 levels did not differ in cerebrospinal fluid between groups, and periodontal status did not affect the levels in any biofluid measured.

**Conclusion:**

Salivary IL-34 is increased in AD patients and is associated with MMSE scores.

**Clinical Relevance:**

Identifying reliable biomarkers for AD is crucial for early detection and intervention. This study suggests that salivary IL-34 could serve as a potential biomarker for AD.

**Supplementary Information:**

The online version contains supplementary material available at 10.1007/s00784-025-06376-4.

## Introduction

It is estimated that about 44 million people worldwide are living with dementia, and due to an aging population, this number could triple by 2050 [1]. Alzheimer’s disease (AD), the most common form of dementia, begins with an asymptomatic phase detectable with biomarkers, then develops into mild cognitive or neurobehavioral impairment, and finally AD dementia [2]. The exact cause of AD remains to be elucidated, however one of the main pathological hallmarks of the disease is the accumulation of protein-complexes in the brain, among them amyloid beta (Ab) plaques and tau protein tangles [3]. Another characteristic of AD is neuroinflammation involving activation of microglial cells and astrocytes, which release pro-inflammatory cytokines and thereby contribute to neurodegeneration [4]. Periodontal diseases have also been linked to an increased risk of developing dementia. [5, 6]. Studies suggest that oral bacteria and/or oral inflammation may contribute to the inflammation and brain changes associated with dementia [7].

The colony stimulating factors colony-stimulating factor 1 (CSF-1) and interleukin-34 (IL-34) bind to their shared receptor CSF-1 receptor (CSF-1R) and are crucial for the proliferation, differentiation, and survival of myeloid cells, including microglia [8]. IL-34 was identified in 2008 as an alternative ligand to CSF-1R and was first believed to be restricted to the skin and central nervous system (CNS) due to the lack of Langerhans cells and microglia in IL-34-deficient mice [9, 10]. IL-34 and CSF-1 levels are altered in several chronic inflammatory diseases, including periodontitis, rheumatoid arthritis and inflammatory bowel disease, making them interesting candidates as potential therapeutic targets or biomarkers of disease [11–13]. The gene expression of CSF-1 and IL-34 in brain tissue during health and cognitive impairment remains to be fully elucidated, however one study reported increased gene expression of CSF-1 and CSF-1R, and reduced expression of IL-34 in human brain samples from AD patients [14].

Identification of reliable biomarkers for AD is crucial for early diagnosis and according to a recent research framework, the diagnosis should be based on biological biomarkers rather than clinical symptoms [15]. The biomarker related methods currently in use for diagnosis of AD are analysis of certain pathogenic proteins, including Ab and tau in cerebrospinal fluid (CSF), along with imaging techniques to identify brain atrophy, measure brain metabolism, and identify accumulation of pathogenic proteins in the brain [16]. In addition, blood-based biomarkers in particular p-tau, are already being tested for clinical routine use[17]. Some studies suggest that periodontal bacteria can enhance the production of Ab. This connection further supports the link between oral health and dementia [18].

Saliva is an easily accessible body fluid, less invasive to collect then CSF. Interestingly, previous experimental models of periodontitis suggest a link between the oral cavity and the brain through various pathways such as nerves, nasal cavity, and the vascular system [19]. Several studies have investigated the potential to reliably identify biological biomarkers for AD in saliva with varying results. In a recent systematic review, the authors concluded that several markers including Aβ_42_, total tau (t-tau), and lactoferrin are promising candidates as salivary biomarkers for early detection of AD [20]. Furthermore, classical markers of inflammation such as IL-1β, tumor necrosis factor-α (TNF-α), and IL-6 have been reported to be decreased in saliva from AD patients [21].

Periodontal disease (PD) can lead to systemic inflammation. This inflammation can affect the brain, contributing to the development and progression of dementia. Elevated levels of inflammatory markers have been found in both periodontal disease and AD [22]. Our research group has previously found increased CSF-1 and lower levels of IL-34 in saliva from periodontal disease patients [23, 24]. The fact that IL-34 and CSF-1 are involved in chronic inflammatory diseases, including AD, along with the notion that there seems to be a connection between the oral cavity and the brain is intriguing. Therefore, we aimed to investigate the salivary levels of IL-34 and CSF-1 in different stages of cognitive impairment, and in relation to periodontal status.

## Material and methods

### Human subjects and collection of biological samples

In this study, a total of 230 individuals were enrolled, including 154 case participants diagnosed with AD (n =52), mild cognitive impairment (MCI, *n = *51), or subjective cognitive impairment (SCI, n =51), and 76 cognitively healthy controls. The case participants were enrolled from the Karolinska Memory Clinic at the Karolinska University Hospital in Huddinge, Sweden, and the cognitively healthy controls were enrolled from the population register in Huddinge, Sweden. AD diagnosis was based on the NIA-AA diagnostic guidelines, MCI according to the Winblad criteria [25], and the SCI in accordance with the pre-MCI SCI criteria [26]. Controls underwent cognitive screening by completing the Mini-mental State Examination (MMSE) and were excluded if having previously sought medical attention for memory loss or had experienced memory loss. All participants underwent a dental examination at the Department of Dental Medicine, Karolinska Institutet, Huddinge, Sweden, including assessment of hard and soft tissues as well as clinical and radiographic periodontal examination. PD was defined as having at least two non-adjacent teeth with marginal alveolar bone loss (MABL) of > 30% and one periodontal pocket of ≥ 6 mm. The enrolment criteria and clinical examinations are thoroughly described in two previous publications from the group [5, 27]. The study was approved by the Regional Ethical Review Board in Stockholm (2012/652-31/1) and conforms to the Declaration of Helsinki standards. Written informed consent was obtained from all study participants.

### Sample collection

In conjunction with the oral examination, venous blood and stimulated whole saliva were collected. Saliva samples were collected after chewing paraffin until 2 ml stimulated saliva was obtained and centrifuged at 500 g for 5 min.

CSF samples were collected as part of the dementia diagnostic workup and lumbar puncture performed in the morning, stored in polypropylene tubes and centrifuged at 3000 rpm for 10 min. After centrifugation, supernatants were collected and stored at −80°C until analysis. CSF was not collected from the controls.

### Analysis of IL-34 and CSF-1 levels

The levels of IL-34 and CSF-1 in stimulated whole saliva, CSF, and plasma were determined by Quantikine enzyme-linked immunosorbent assays according to the manufacturer’s instructions (R&D Systems, Minneapolis, MN, USA). The assays have previously been validated for saliva by our laboratory [23]. Readings were made with a microplate spectrophotometer (SpectraMax 340, Sunnyvale, CA, USA) with a wavelength of 450 nm and correction set to 540 nm for background subtraction. Samples below the detection limit were set to 0. The detection of IL-34 was 97.8% in saliva, 97.8% in CSF, and 35.4% in plasma. The detection of CSF-1 was 99.6% in saliva, 92% in CSF, and 98.2% in plasma.

### Data analysis

Statistical analysis was made using GraphPad Prism 10 (GraphPad Software Inc., La Jolla, CA) and Statistical Package for Social Sciences (SPSS), version 29 (IBM Corporation, Armonk, NY, USA). Mann-Whitney or Kruskal-Wallis with Dunn-Bonferroni post-hoc test was used to analyze differences between groups. Spearman’s rank test was used to determine correlations between IL-34 and CSF-1 in saliva, CSF, and plasma. Multiple linear regression was done with IL-34 and CSF-1 as dependent variables and number of pockets ≥ 6 mm, gender, age, diabetes, smoking, and MMSE as independent variables. Cytokines were log-transformed before inclusion into the model. Statistical significance was set at *p <* 0.05.

## Results

### Clinical characteristics of the study participants

The clinical characteristics of the study participants are presented in Table 1. Age and MMSE score differed between the groups (*p <* 0.010). The AD, MCI, and SCI groups had a higher number of periodontal pockets 4–5 mm and ≥ 6 mm compared with controls (*p <* 0.010). No differences among the groups were observed for sex, diabetes, smoking, number of teeth, and marginal bone loss.

**Table 1:** Clinical characteristics of the study groups

Variables	Controls (*n = *76)	AD (*n = *52)	MCI (*n = *51)	SCI (*n = *51)	*p*-value
Age, n	69.5 (8.4)	71.3 (11.2)^b^	70.1 (10.7)^b^	60.7 (24.2)^a^	*<0.010*
Gender, n (male)	33	24	26	22	0.832
MMSE score	29.5 (1.0)	25.0 (6.0)^abc^	28.0 (3.0)^ab^	30.0 (1.0)^a^	*<0.010*
Diabetes, n	8	7	4	6	0.827
BMI	26.5 (5.5)	23.6 (4.9)^ab^	24.8 (4.1)^a^	25.4 (7.4)	*<0.010*
Smoking					
Current, n	6	6	2	2	0.723
Previous, n	32	22	24	20	
Never, n	38	24	25	29	
Nb of teeth	27.0 (4)	26.0 (6.0)^b^	26.0 (4.0)^b^	27.0 (4.0)	0.052
Nb of pockets					
Sites with PPD 4–5 mm	4.0 (7.0)	10.5 (11.0)^a^	11.0 (13.0)^a^	10.0 (14.0)^a^	*<0.010*
Sites with PPD ≥ 6 mm	0.0 (0.0)	2.0 (4.0)^abc^	0.0 (3.0)^a^	0.0 (3.0)^a^	*<0.010*
BOP, %	16.5 (15.0)	21.0 (21.0)^a^	29.0 (17.0)^a^	23.0 (16.0)^a^	*<0.010*
Marginal alveolar bone loss					
No or mild, n	50	27	29	32	0.137
Localized, n	24	17	20	16	
Generalized, n	2	8	2	4	

Data is presented as median and interquartile range or frequencies. Abbreviations: *MMSE* mini mental state examination; *BMI* body mass index, *PPD* probing pocket depth, *BOP* bleeding on probing. Analysis was performed using Chi-square test or Fisher’s exact test for categorical variables. Kruskal-Wallis was used for continuous variables. ^a^*p<*0.05 compared to control group. ^b^*p<*0.05 compared to SCI. ^c^*p<*0.05 compared to MCI

### IL-34 and CSF-1 levels in saliva, CSF, and plasma in different stages of cognitive impairment

**Fig 1:**
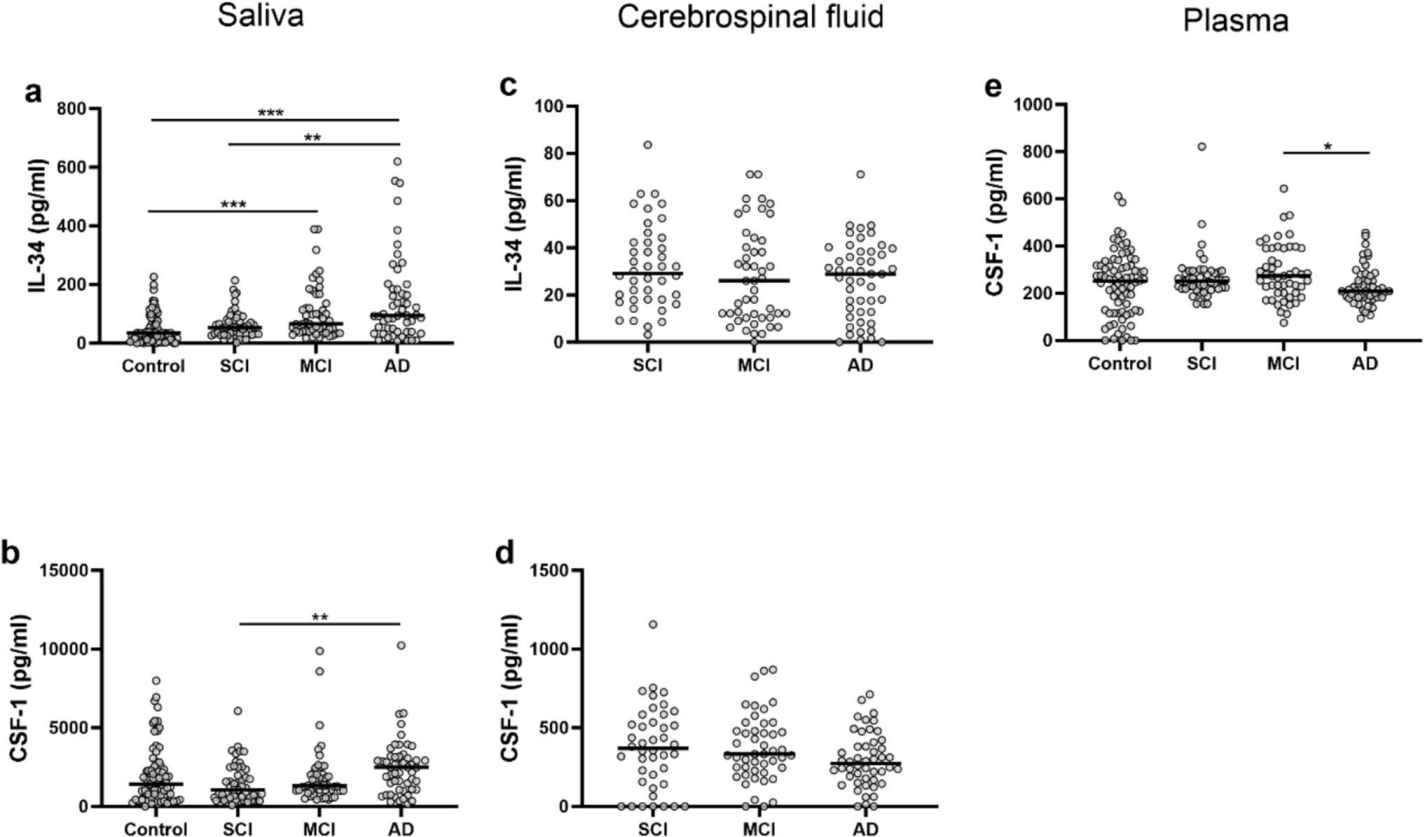
IL-34 and CSF-1 concentrations in saliva, cerebrospinal fluid (CSF) and plasma in different stages of cognitive impairment. Concentrations of (**a**) IL-34 in saliva, and (**b**) CSF-1 in saliva, from controls (*n=* 76), subjects with subjective cognitive impairment (SCI, *n=* 51), subjects with mild cognitive impairment (MCI, *n=* 51), and subjects suffering from Alzheimer’s disease (AD, *n=* 52). (**c**) IL-34 in CSF and (**d**) CSF-1 in CSF from SCI (*n=* 42), MCI (*n=* 47) and AD (*n=* 48). (**e**) CSF-1 in plasma from controls (*n=* 76), SCI (*n=* 49), MCI (*n=* 50), and AD (*n=* 51). Data for IL-34 in plasma not presented due to low detectability. *P*-values were determined using Kruskal-Wallis with Dunn-Bonferroni post-hoc test, *p<0.05, **p<0.01, p***<0.001

We analyzed the levels of IL-34 and CSF-1 in saliva, CSF, and plasma in participants with different stages of cognition impairment. Salivary IL-34 levels were increased in AD compared with SCI (*p =* 0.010) and controls (*p <* 0.001), as well as in MCI compared with controls (*p <* 0.001, Fig. 1A). In AD patients, the concentration of CSF-1 in saliva was elevated compared with SCI (*p =* 0.003), but no difference was observed when compared with controls (Fig. 1B).

The levels of IL-34 and CSF-1 in CSF were measured in SCI, MCI, and AD, as CSF samples were not collected from the control group. No differences in IL-34 and CSF-1 levels were observed in CSF between the groups (Fig. 1C, D). Analysis of CSF-1 in plasma revealed lower levels in AD patients compared with MCI (*p =* 0.040), while no difference was observed in comparison with control or SCI groups (Fig. 1D). IL-34 levels were generally under the detection limit in plasma samples and therefore were not used in the comparison between groups.

### IL-34 and CSF-1 levels in saliva, CSF, and plasma from non-periodontitis and periodontitis subjects

**Fig 2:**
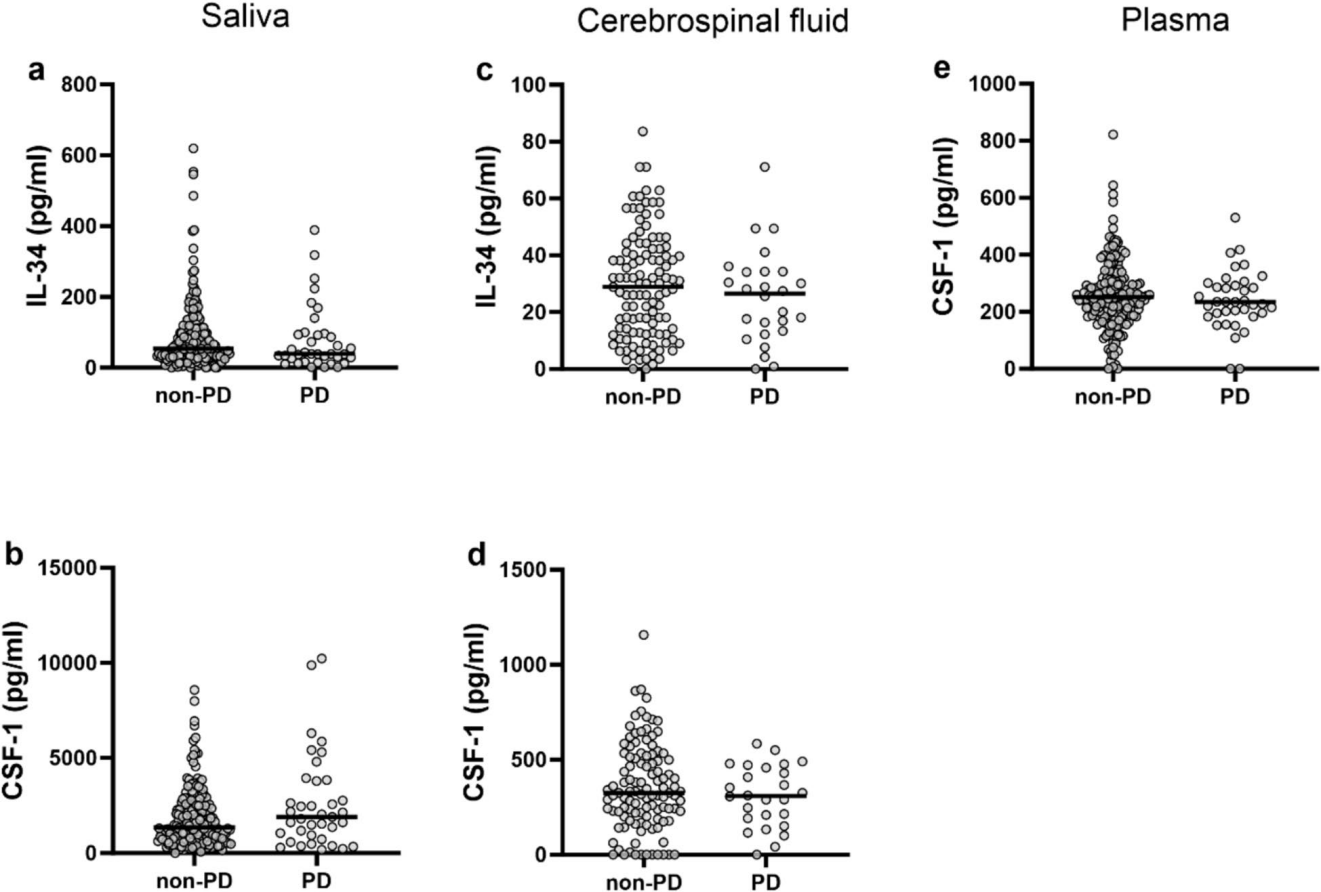
IL-34 and CSF-1 concentrations in saliva, cerebrospinal fluid (CSF) and plasma from non-periodontitis and periodontitis subjects (PD). Concentrations of (**a**) IL-34 in saliva, and (**b**) CSF-1 in saliva from non-PD (*n=* 193), and PD (*n=* 37), (**c**) IL-34 in CSF, and (**d**) CSF-1 in CSF from non-PD (*n=* 111) and PD (*n=* 26), (**e**) CSF-1 in plasma from non-PD (*n=* 189) and PD (*n=*37). Data for IL-34 in plasma is not presented due to low detectability. *P*-values were determined by Mann-Whitney *U* test

The study included 37 PD patients and 193 non-PD patients. To investigate if PD affected the levels of IL-34 and CSF-1 in the different fluids, we compared PD and non-PD patients and found no differences between the groups (Fig. 2A-E). We also assessed if MABL affected the levels of IL-34 and CSF-1, but there were no differences between individuals with no/mild MABL, localized MABL, and generalized MABL (Sup. Figure 1).

### Correlation of IL-34 and CSF-1 in saliva, CSF, and plasma

**Fig 3:**
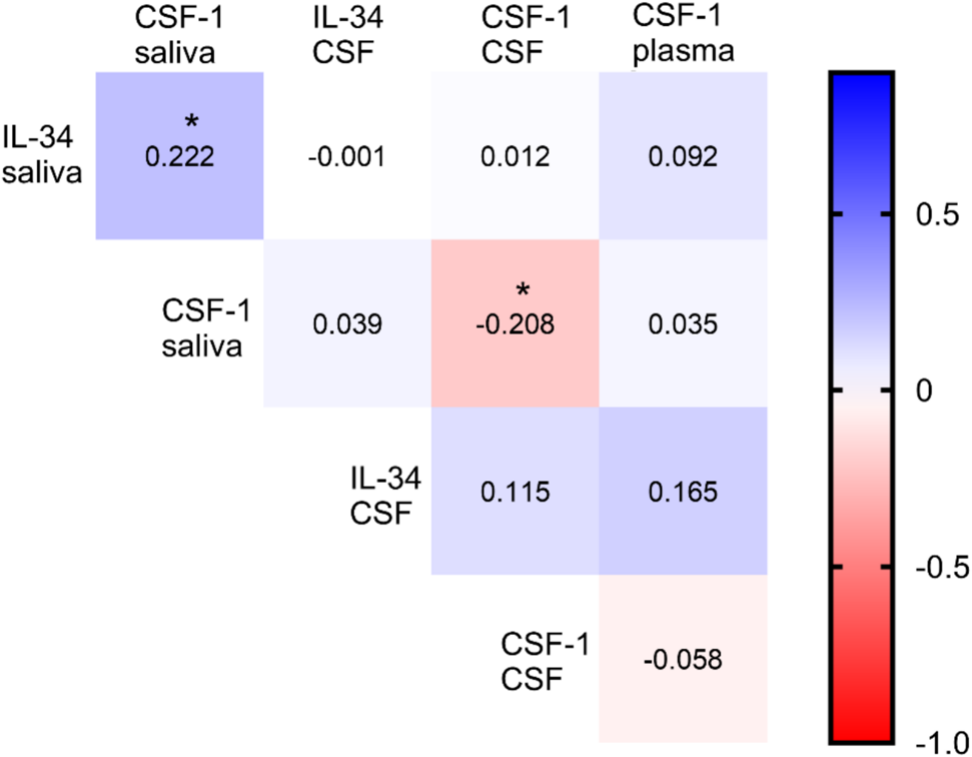
Correlation heat map of IL-34 and CSF-1 in saliva, cerebrospinal fluid (CSF), and plasma. Spearman correlation was used for analysis. Values within the squares indicate the correlation coefficient. The asterisk indicates significant correlation

Correlations between IL-34 and CSF-1 in saliva, CSF and plasma are presented in Fig. 3. IL-34 in saliva correlated with CSF-1 in saliva (r = 0.222). Additionally, a negative correlation was observed between CSF-1 in saliva and CSF-1 in CSF (r = −0.208).

### Association of IL-34 and CSF-1 in saliva, CSF, and plasma with clinical parameters

A regression analysis was performed to investigate associations between IL-34 and CSF-1 in saliva, CSF, and plasma with clinical parameters (Table 2). The clinical parameters included in the model were age, gender, MMSE, diabetes, BMI (body mass index), smoking, and number of pockets of ≥ 6 mm. Salivary IL-34 levels were associated with MMSE score and BMI (*p =* 0.002 and *p =* 0.042, respectively). Salivary CSF-1 levels were associated with age (*p =* 0.015), but not with any of the other clinical parameters. In CSF, we noted a tendency towards an association between IL-34 and MMSE score (*p =* 0.087), whereas no associations were found between CSF-1 and the clinical parameters. CSF-1 in plasma was not associated with any of the clinical parameters.

**Table 2:** Association of IL-34 and CSF-1 in saliva, CSF, and plasma with clinical parameters

Variables	Coefficient (β)	95% CI	*p*-value
IL-34 in saliva			
Age (years)	0.004	−0.005 – 0.01	0.386
Gender	−0.02	−0.16 – 0.11	0.734
MMSE	−0.04	−0.07 – −0.01	*0.002*
Diabetes	−0.01	−0.23 – 0.21	0.935
BMI	−0.02	−0.04 – −0.001	*0.042*
Smoking	0.002	−0.11 – 0.11	0.972
Pockets≥6 mm	−0.004	−0.02 – 0.01	0.519
CSF-1 in saliva			
Age (years)	0.01	0.002–0.02	*0.015*
Gender	0.02	−0.01 – 0.13	0.778
MMSE	−0.002	−0.02 – 0.02	0.830
Diabetes	0.03	−0.16 – 0.22	0.760
BMI	−0.002	−0.02 – 0.01	0.815
Smoking	0.08	−0.01 – 0.17	0.092
Pockets≥6 mm	0.01	−0.002 – 0.02	0.135
IL-34 in CSF			
Age (years)	0.001	−0.01 – 0.01	0.895
Gender	0.03	−0.10 – 0.16	0.659
MMSE	0.02	−0.003 – 0.04	0.087
Diabetes	−0.22	−0.46 – 0.03	0.083
BMI	0.001	−0.02 – 0.02	0.880
Smoking	0.05	−0.07 – 0.16	0.432
Pockets≥6 mm	−0.005	−0.02 – 0.007	0.432
CSF-1 in CSF			
Age (years)	0.01	−0.005 – 0.03	0.163
Gender	−0.16	−0.42 – 0.01	0.225
MMSE	−0.01	−0.05 – 0.03	0.591
Diabetes	0.17	−0.30 – 0.64	0.477
BMI	0.01	−0.02 – 0.05	0.426
Smoking	0.005	−0.021 – 0.22	0.960
Pockets≥6 mm	0.003	−0.02 – 0.03	0.825
CSF-1 in plasma			
Age (years)	0.004	−0.004 – 0.01	0.309
Gender	−0.02	−0.12 – 0.09	0.778
MMSE	−0.01	−0.03 – 0.01	0.538
Diabetes	−0.03	−0.20 – 0.14	0.711
BMI	0.00	−0.01 – 0.01	0.951
Smoking	−0.04	−0.13 – 0.04	0.304
Pockets≥6 mm	0.00	−0.01 – 0.01	0.921

Linear regression analyses of IL-34 and CSF-1 in saliva, CSF, and plasma. Variables entered in the model; age, gender, MMSE, diabetes, BMI, smoking, and number of pockets ≥ 6 mm. Cytokine levels were log transformed

## Discussion

Identification of biomarkers for early detection of AD is crucial to detect the disease before symptoms such as cognitive impairment and personality changes arise. In this study, we investigated the levels of the macrophage growth factors IL-34 and CSF-1 in three biofluids at different stages of cognitive impairment and found higher levels of IL-34 in saliva from AD patients compared with controls and subjects with SCI. The levels of IL-34 in saliva were also higher in MCI cases compared to controls. Furthermore, IL-34 levels in saliva associated negatively with MMSE scores. Salivary CSF-1 levels were slightly elevated in AD patients, but only statistically significantly in comparison with SCI.

In plasma, CSF-1 levels were lower in AD patients compared with MCI. The study’s clinical characteristics reveal significant differences in age and MMSE scores between groups, indicating varying levels of cognitive impairment. Notably, the AD, MCI, and SCI groups exhibited more periodontal pockets (of 4–5 mm and ≥ 6 mm) compared to controls. This suggests a potential link between periodontal disease and cognitive decline, as these groups had more severe periodontal conditions than controls as previously reported [5].

The use of saliva as a diagnostic tool for AD has gained attention lately. The rationale for using saliva is the less invasive collection method as compared to CSF and blood samples. The CSF biomarkers in use for AD diagnostics are primarily Aβ_42_, phosphorylated (p)-tau and t-tau. Studies have reported altered levels of AD biomarkers in saliva, where the most studied is Aβ_42_ [20, 28, 29]. The pathway for these biomarkers from the CNS to saliva has not yet been clarified. However, the salivary glands are innervated by the autonomic nervous system, which controls the flow and composition of saliva. Thus, AD-induced alterations in the autonomic nervous system could change the composition of saliva. Furthermore, biomarkers can pass from the blood to the saliva and it has also been suggested that cells in the salivary glands can produce certain AD-biomarkers [30].

Identification of salivary biomarkers that are specific for AD is difficult due to the multiple factors that can affect the salivary composition, including PD and other systemic diseases [31, 32]. For the measurement of cytokine levels in this study, we used immunoassays that have previously been validated for analysis of saliva. Interestingly, we have previously reported lower salivary IL-34 levels in PD patients compared with controls, suggesting that IL-34 in saliva is not increased by PD inflammation [23]. Here, we found an increase in salivary IL-34 levels in MCI compared with controls, suggesting that it may be indicative of cognitive impairment before the onset of AD. In AD, the levels of IL-34 in saliva were higher than those in MCI and compared with controls, thus an increase was found with the IL-34 levels increasing from controls < SCI < MCI < AD. Compelling evidence suggests that blood-brain barrier (BBB)-dysfunction plays an important role in the pathogenesis of AD, resulting in leakage of inflammatory mediators and infiltration of peripheral immune cells into the neurovascular space [33]. IL-34 is produced by neurons in the CNS and has been reported to induce neuroprotective properties of microglia, as well as protecting the BBB integrity by up-regulating tight junction proteins in CSF-1R-expressing endothelial cells [34]. On the other hand, some studies have described an association between cognitive impairment and decreased serum levels of IL-34, particularly in vascular dementia. This indicates that IL-34 might have a protective role in maintaining cognitive function [35]. Although more research is needed on the subject, one could speculate that the elevated levels of IL-34 in saliva from AD patients stem in part from increased production of IL-34 due to a dysfunctional BBB.

In the regression analysis, we observed an association between salivary IL-34 and BMI. The reason for this association remains to be fully elucidated, however elevated IL-34 levels have previously been reported in gingival crevicular fluid (GCF) and in plasma from obese PD patients compared to non-obese PD patients and controls [36]. An explanation for this could be an overall increased inflammatory state in obese patients [37].

Elevated salivary IL-34 and CSF-1 levels in AD and MCI patients suggest an inflammatory component linked to cognitive decline. However, to our surprise, we found unaltered levels of IL-34 in saliva when comparing PD and non-PD subjects. This lack of differences indicates that PD may not directly affect this marker. However, others have reported elevated levels of IL-34 in gingival crevicular fluid (GCF) and serum from PD-patients [38], suggesting an involvement of IL-34 in periodontal inflammation. It is reasonable to believe that this discrepancy is due to the sources of sampling, specifically saliva versus gingival pockets and to the sampling techniques. However, more studies are needed to elucidate the expression patterns of IL-34 in oral biofluids. In the present study, increased salivary CSF-1 levels were associated with older age, which is in line with previous results from our group [39], whereas the CSF-1 levels in PD were unchanged compared with the non-PD group, which is not in line with our previous observation [23]. One reason for this discrepancy may be the difference in size of the groups (37 PD participants and 193 non-PD), because subjects were enrolled in the study on the basis of cognitive diagnosis and not based on periodontal status. One could also speculate that the presence of periodontal inflammation in the AD and MCI groups could mask differences in biomarker expression deriving from cognitive dysfunction and perhaps medication.

In our study, the levels of IL-34 in plasma were in most cases under the detection limit. Previous studies have reported increased IL-34 levels in plasma in PD and systemic inflammatory conditions, such as rheumatoid arthritis [36, 40]. The immunoassay used in this study is validated for analysis of plasma, and it is possible that the low detectability of IL-34 is the result of low plasma levels of IL-34 in the study groups. A limitation of the study is that we only used stimulated saliva since it is a more time efficient and easier sampling method for the patients compared to the collection of unstimulated saliva. Indeed, it has been shown that the collection method can have an impact on the levels of salivary biomarkers in AD patients [41]. However, our group has previously reported no difference in salivary IL-34 levels between stimulated and unstimulated saliva [23]. It should be noted that we did not measure the salivary flow rate, and this should be taken into consideration for future studies since compromised salivary flow is common in AD patients [42]. Another point to consider is the possible effects of medications taken by the patients, which could have an impact on salivary flow and composition. We believe, however, that the assessment of three different biofluids, namely saliva, CSF and plasma, along with the thorough clinical and radiographic oral examinations of the patients, adds considerable strength to the study.

In conclusion, this study reports elevated IL-34 levels in saliva from patients suffering from AD and an inverse association between salivary IL-34 and MMSE score. Although more investigations are needed, this suggests that IL-34 may be a potential salivary biomarker for AD and could perhaps be used as an additional disease indicator.

## Supplementary Information

Below is the link to the electronic supplementary material.

Supplementary file1 (DOCX 293 KB)

## Data Availability

The data supporting the results in this study are available from the corresponding author upon reasonable request.
