# Supplementary material for: Elevated levels of salivary interleukin-34 in patients suffering from Alzheimer’s disease
Source: Clin Oral Investig. 2025 May 20;29(6):303. doi: 10.1007/s00784-025-06376-4 (PMC12089168; doi:10.1007/s00784-025-06376-4)
Supplement: Supplementary file 1 — Supplementary file1 (DOCX 293 KB) [file 784_2025_6376_MOESM1_ESM.docx]

**Supplementary figures**

**
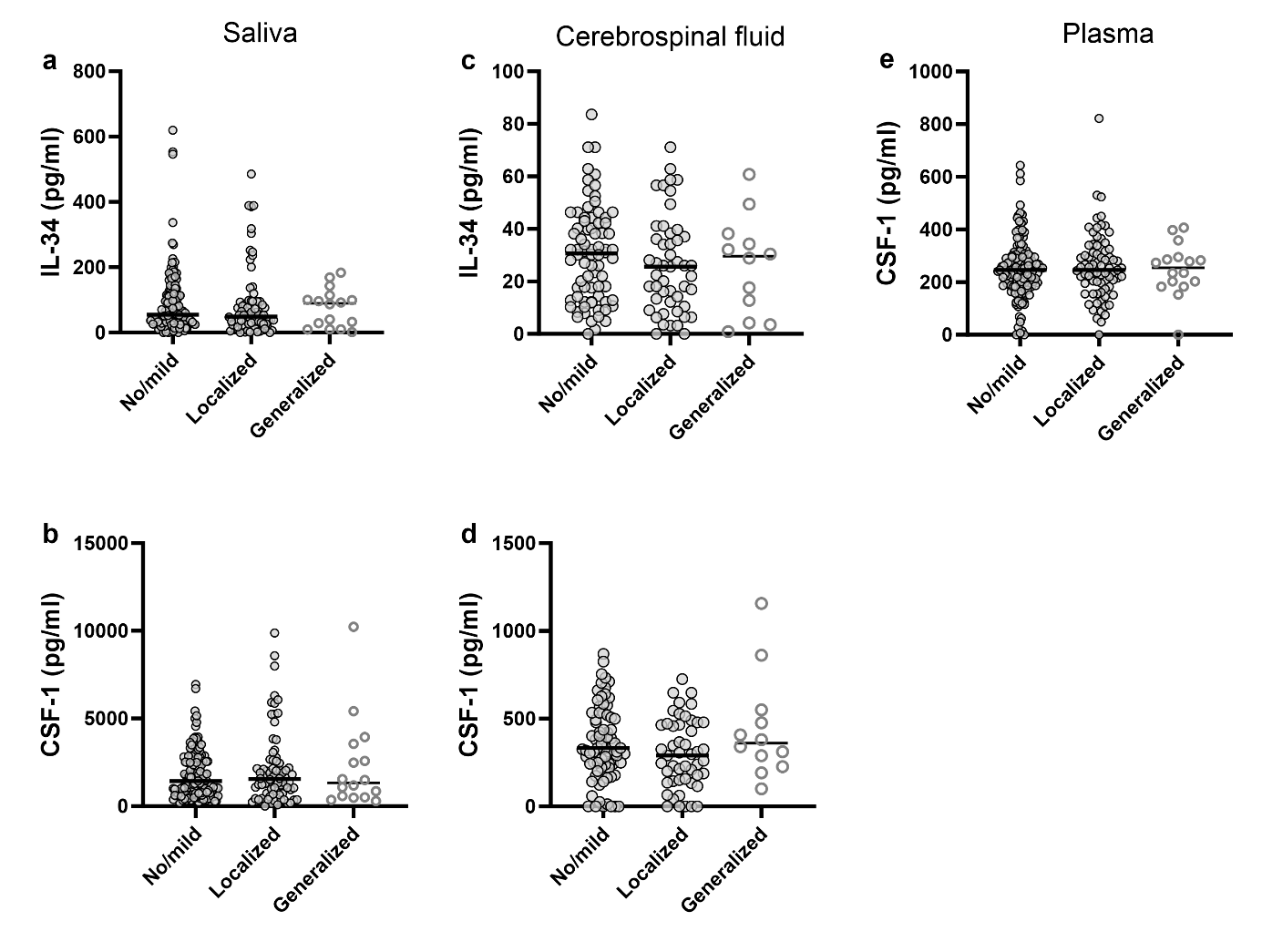
**

**Supplementary fig 1. IL-34 and CSF-1 concentrations in saliva, cerebrospinal fluid in relation to marginal alveolar bone loss.**

Concentrations of **a.** IL-34 in saliva, and **b.** CSF-1 in saliva from individuals with no/mild MABL (n= 138 ), localized MABL (n= 76) and generalized MABL (n= 16), **c.** IL-34 in CSF, and **d.** CSF-1 in CSF, no/mild MABL (n= 76), localized MABL (n= 49), generalized MABL (n= 12) **e.** CSF-1 in plasma, no/mild MABL (n= 135), localized MABL (n= 75), generalized MABL (n= 16). Data for IL-34 in plasma not presented due to low detectability. P-values were determined by Kruskal-Wallis with Dunn-Bonferroni post-hoc test.
